# Supplementary material for: A white-to-opaque-like phenotypic switch in the yeast Torulaspora microellipsoides
Source: Commun Biol. 2020 Feb 28;3:86. doi: 10.1038/s42003-020-0815-6 (PMC7048803; doi:10.1038/s42003-020-0815-6)
Supplement: Supplementary file 1 — Supplementary Information [file 42003_2020_815_MOESM1_ESM.pdf]

Supplementary Figures

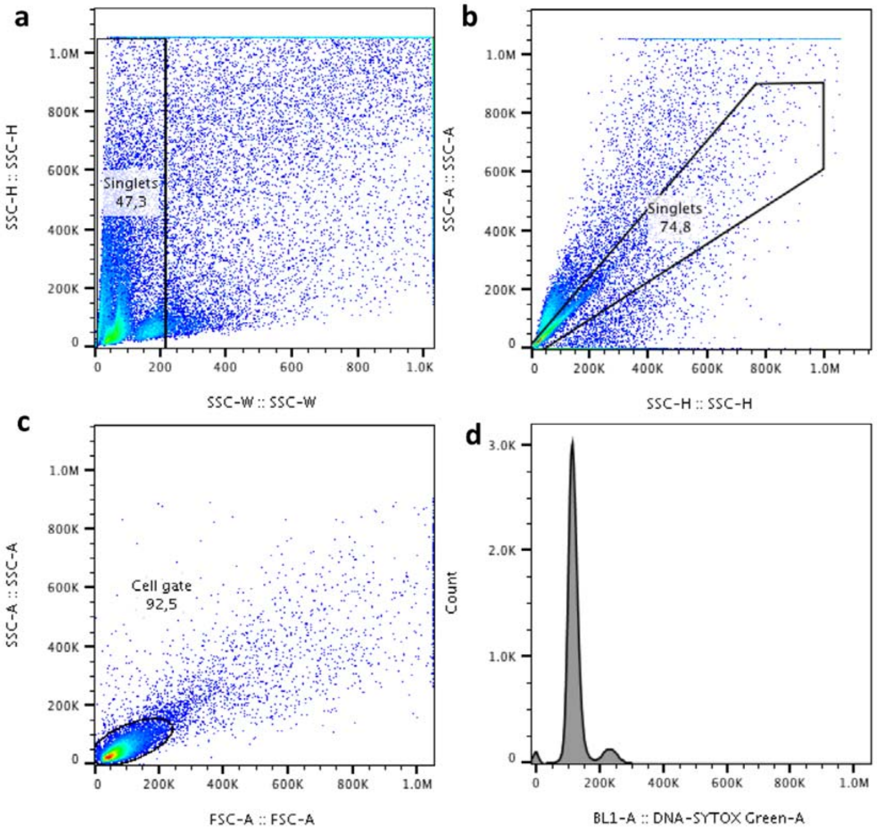

Supplementary Figure 1. Flow cytometry gating strategy for DNA content analysis of *T. microellipsoides* isolates. Example shown is for WT white cells.

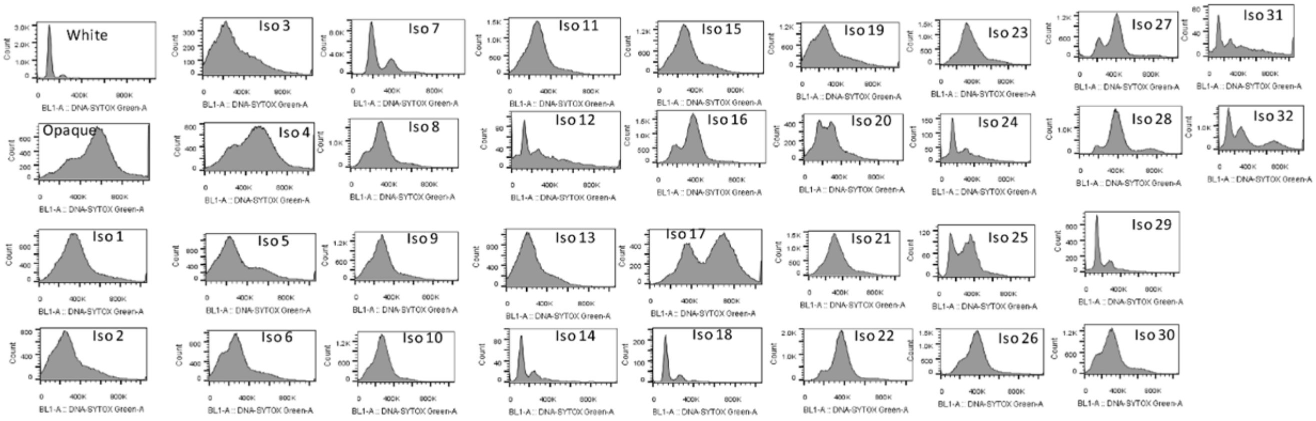

Supplementary Figure 2. Ploidy analysis of white, opaque, and 32 opaque-derived isolated of *T. microellipsoides*, as measured by flow cytometry.

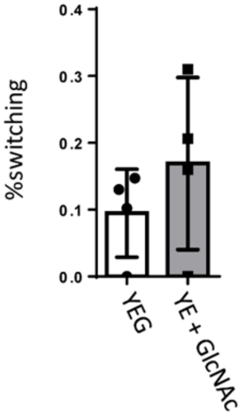

Supplementary Figure 3. White to opaque switching rate of WT cells grown on either YEG or YEG + 2% N-acetyl glucosamine (GlcNAc). Error bars represent the standard deviation of the mean. N=3 independent experiments. Error bars represent the standard deviation of the mean; significance values are shown in Supplementary Data 1.

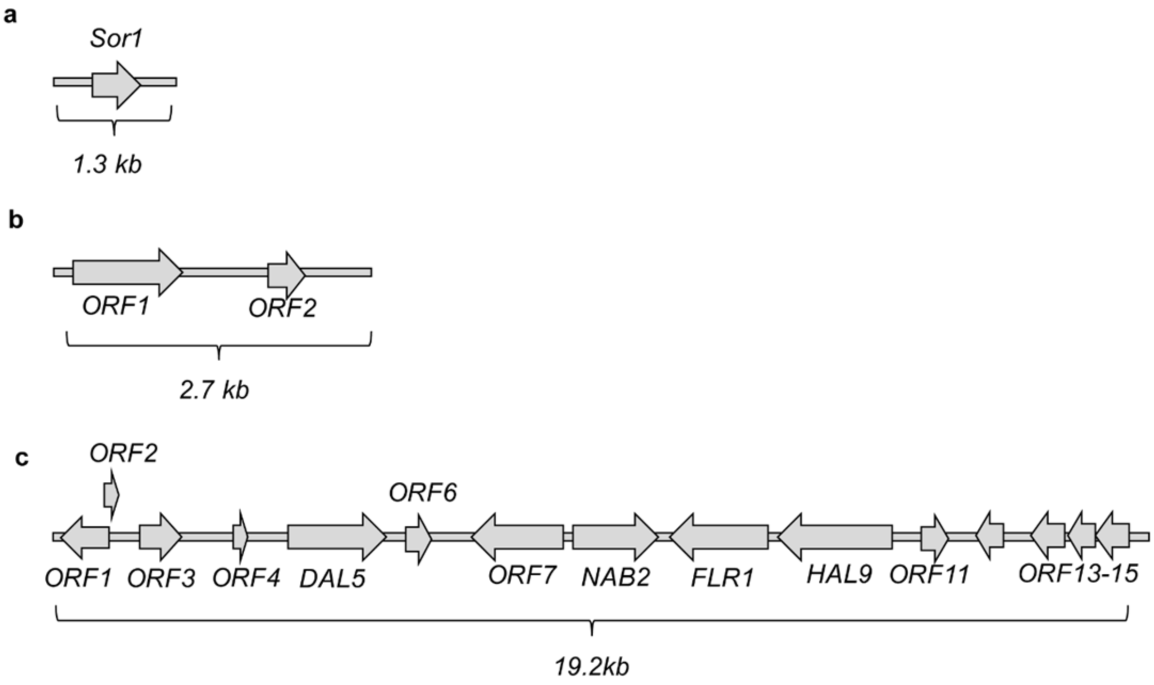

Supplementary Figure 4. Schematic representation of putatively translocated regions in the genome of opaque cells compared to white cells. **a,** Schematic map of the 1.3 kb translocated region between white and opaque cells. **b,** Schematic map of the 2.7 kb translocated region between white and opaque cells **c,** Schematic map of the 19.2 kb putative translocated region between white and opaque cells. Annotations are based off alignment to the *S. cerevisiae* or *C. alibcans* homologues. Un-annotated ORF's are those for which a homologue was not detected. DAL5 denotes "degradation of Allantoin 5", NAB2 denotes "Nuclear polyAdenylated RNA-binding 2", FLR1 denotes "Fluconazole Resistance 1", and HAL9 denotes "HALotolerance 9".

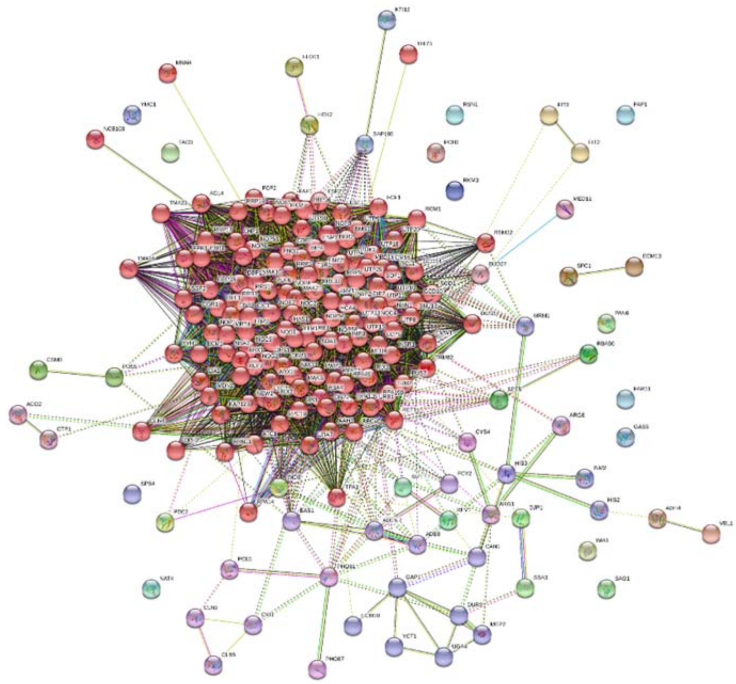

**Biological Process**		
**decription**	**count in gene set**	**false discovery rate**
ribosome biogenesis	112 of 403	9.92x10^-75
ribonucleoprotein complex biogenesis	113 of 485	1.24x10^-68
rRNA metabolic process	92 of 320	5.48x10^-60
ncRNA metabolic process	104 of 494	4x10^-58
rRNA processing	86 of 277	7.02x10^-58
**Molecular function**		
**decription**	**count in gene set**	**false discovery rate**
RNA binding	75 of 669	2.47x10^-22
snoRNA binding	20 of 26	2.09x10^-17
nucleic acid binding	87 of 1234	1.92x10^-14
mRNA binding	31 of 188	4.13x10^-12
heterocyclic compound binding	102 of 1925	6.89x10^-10
**Cellular Component**		
**decription**	**count in gene set**	**false discovery rate**
nucleolus	105 of 308	3.05x10^-77
preribosome	77 of 182	1.82x10^-59
nuclear lumen	119 of 855	8.4x10^-52
intracellular organelle lumen	123 of 1180	1.91x10^-41
90S preribosome	43 of 90	1.34x10^-33
**KEGG Pathways**		
**decription**	**count in gene set**	**false discovery rate**
ribosome biogenesis in eukaryotes	30 of 73	2.19x10^-21
RNA polymerase	8 of 30	0.00017
purine metabolism	13 of 99	0.0002
pyrmidine metabolism	9 of 71	0.004
**Reactome pathways**		
**decription**	**count in gene set**	**false discovery rate**
major pathway or rRNA processing in the nucleolus nad cytoplasm	26 of 56	6.62x10^-19
metabolism of RNA	30 of 271	4.01x10^-8
RNA polymerase I transcription	4 of 11	0.0197

Supplementary Figure 5. Graphical representation of STRING-analysis of genes upregulated in opaque compared to white cells, shown on the left. Shown on the right is a table of biological and molecular functions enriched in this data set, as cell as cellular components and KEGG pathways.

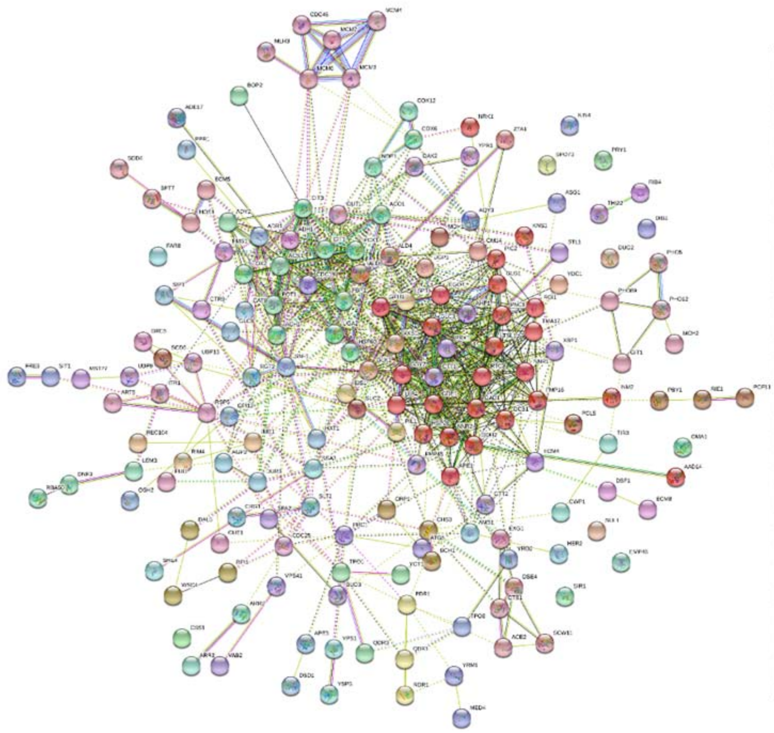

Biological Process		
**decription**	**count in gene set**	**false discovery rate**
carbohydrate metabolic process	30 of 252	3.4x 10^-8
cellular carbohydrate metabolic process	21 of 163	6.43X10^-6
oxidation-reduction process	34 of 445	2.35x10^-5
carbohydrate catabolic process	13 of 84	0.00041
polysaccharide metabolit process	13 of 89	0.00059
**Molecular function**		
**decription**	**count in gene set**	**false discovery rate**
catalytic activity	92 of 2212	0.00014
transporter activity	29 of 415	0.00063
transmembrane transporter activity	25 of 347	0.0013
secondary active transmembrane transport	12 of 89	0.0013
hydrolase activity, O-glycosyl comounts	9 of 48	0.0013
**Cellular Component**		
**decription**	**count in gene set**	**false discovery rate**
cell periphery	55 of 790	7.06x10^-9
plasma membrane	40 of 528	4.24x10^-7
cell	167 of 5412	9.38x10^-5
cell part	166 of 5410	0.00023
CMG complex	5 of 11	0.0022
**KEGG Pathways**		
**decription**	**count in gene set**	**false discovery rate**
starch and sucrose metabolism	10 of 40	2.75x10^-5
metabolic pathways	38 of 716	0.0011
biosynthesis of secondary metabolites	20 of 295	0.0036
glycolysis/gluconeogenesis	8 of 55	0.0036
carbon metabolism	11 of 112	0.004

Supplementary Figure 6. Graphical representation of STRING-analysis of genes upregulated in white compared to opaque cells, showing known or predicted protein-protein interactions, shown on the left. Shown on the right is a table of biological and molecular functions enriched in this data set, as cell as cellular components and KEGG pathways.

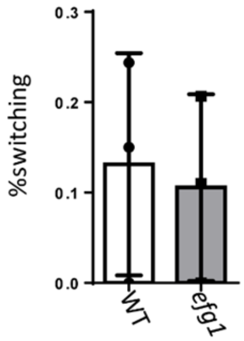

Supplementary Figure 7. White to opaque switching rate of WT and efg1 deletion strain, on either standard YEG plates. N=3 independent experiments. Error bars represent the standard deviation of the mean; significance values are shown in Supplementary Data 1.

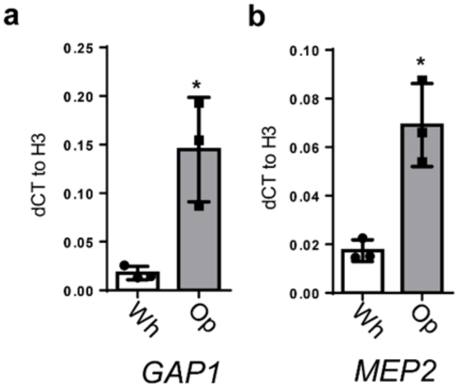

Supplementary Figure 8. Expression analysis of the homologues of **a,** *GAP1* and **b,** *MEP2* in *T. microellipsoides* white and opaque cells, as measured RT-qPCR. N=3 independent cultures. Error bars represent the standard deviation of the mean; significance values are shown in Supplementary Data 1.

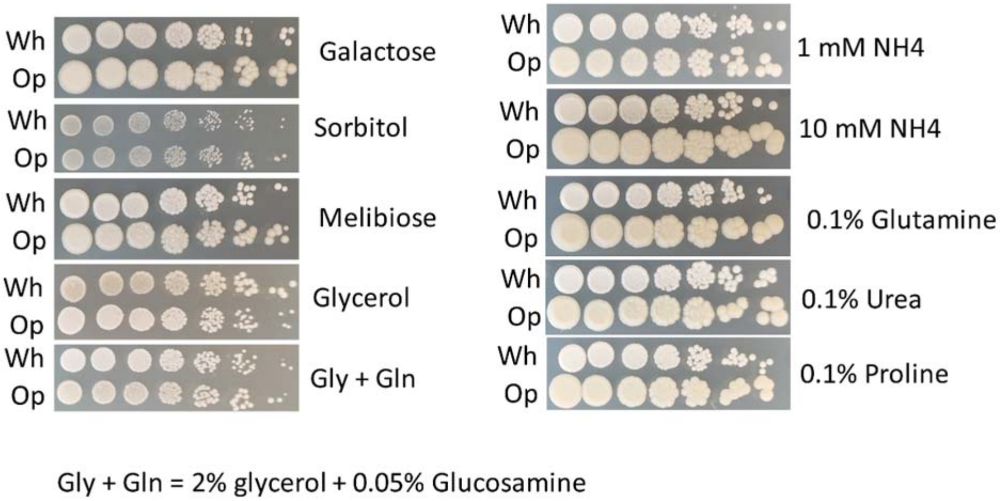

Supplementary Figure 9. Growth of white and opaque cells on YNB + various carbon and nitrogen sources, assayed by dilution plating.

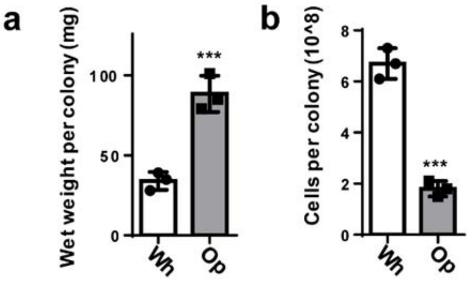

Supplementary Figure 10. a, quantification of colony mass of white and opaque cells by measurements of wet weight of single colonies. **b,** Quantification of number of cells per colony of white and opaque cells. N=3 independent colonies. Error bars represent the standard deviation of the mean; significance values are shown in Supplementary Data 1.

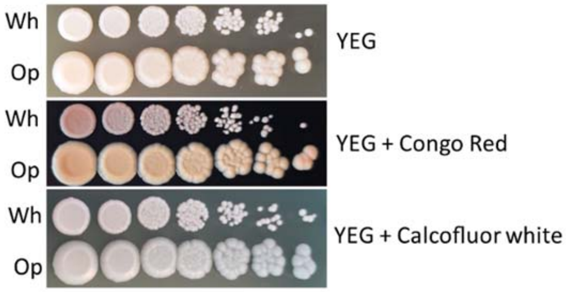

Supplementary Figure 11. Growth of white and opaque cells on YEG, YEG + Congo Red, and YEG + Calcofluor white, as assay by dilution plating.

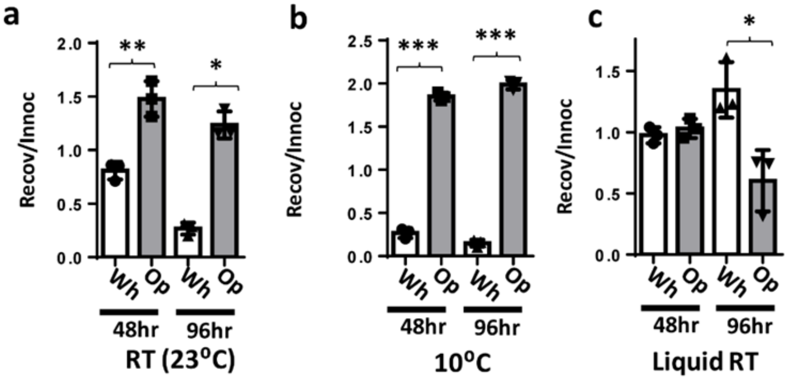

Supplementary Figure 12. a, Relative competitive fitness of white and opaque cells grown on agar plates at room temperature after 48 and 96 hours. **b,** Relative competitive fitness of white and opaque cells grown in agar plates at 10 □C after 48 and 96 hours. **c,** Relative competitive fitness of white and opaque cells grown in liquid YEG after 96 hours. N=3 independent experiments. Error bars represent the standard deviation of the mean; significance values are shown in Supplementary Data 1.

Supplementary Figure 13. Un-cropped gel image of Figure 1f.

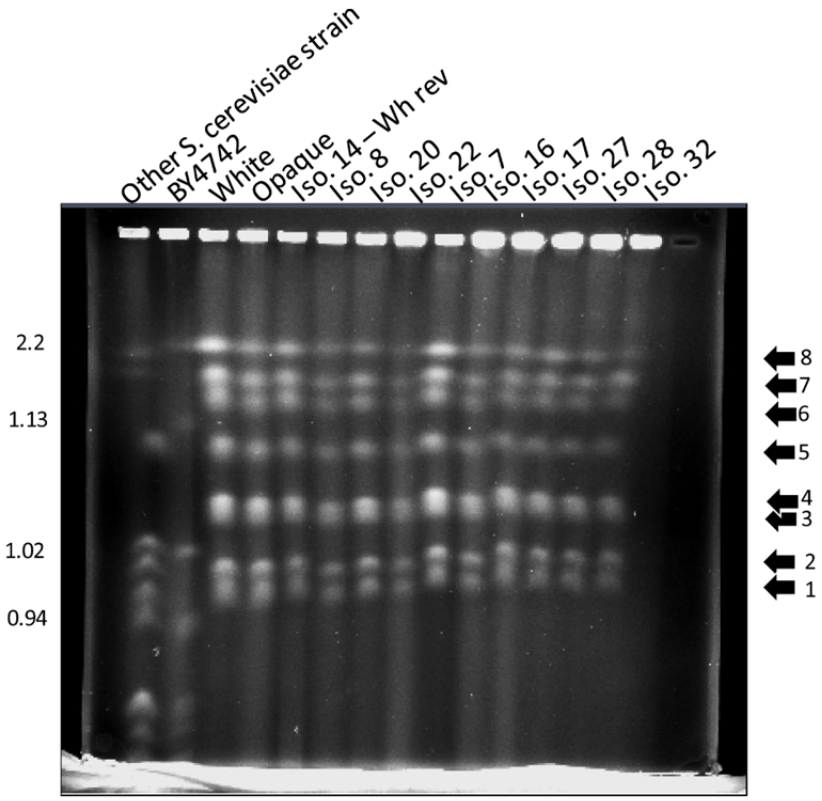

Supplementary Tables

Supplementary Table 1. Putative translocated regions in opaque cells compared to white cells.

	**Gene homologue**	**Putative function(s)**	**Differential expression between white and opaque? (log2-fold change 2 or greater)**
**Region A (1.3 kb)**	SOR1	Sorbitol dehydrogenase	No
**Region B (2.7 kb)**	ORF1	No identifiable homologue	No
	ORF2	No identifiable homologue	No
**Region**	ORF1	No identifiable homologue	No

**C (19.2 kb)**			
	ORF2	No identifiable homologue	No
	ORF3	No identifiable homologue	No
	ORF4	No identifiable homologue	No
	DAL5	Allantoate permease; ureidosuccinate permease; transports dipeptides	No
	ORF6	No identifiable homologue	No
	ORF7	No identifiable homologue	No
	NAB2	Nuclear polyadenylated RNA-binding protein; required for nuclear mRNA export and poly(A) tail length control; stimulates RNA polymerase III transcription by enhancing TFIIIB binding to promoters; protects mRNA against decay by the nuclear exosome in a poly(A)-tail-dependent manner; involved in forming export-competent mRNPs in the nucleus; autoregulates mRNA levels; NLS binds Kap104p	No
	FLR1	Plasma membrane transporter of the major facilitator superfamily; member of the 12-spanner drug:H(+) antiporter DHA1 family; involved in efflux of fluconazole, diazaborine, benomyl, methotrexate, and other drugs	No
	HAL9	Putative transcription factor containing a zinc finger; overexpression increases salt tolerance through increased expression of the ENA1 (Na+/Li+ extrusion pump) gene while gene disruption decreases both salt tolerance and ENA1 expression	No
	ORF11	No identifiable homologue	No
	ORF12	No identifiable homologue	No
	ORF13	No identifiable homologue	No
	ORF14	No identifiable homologue	No

	ORF15	No identifiable homologue	No

Supplementary Table 2. Primers, plasmids and strains used in this study.

**Systematic name**	**Specific use**	**Primer sequence (5' to 3')**
CB276	*T. microellipsoides* Histone H3 forward	CATCTGCTATTGGTGCTTTGC
CB277	*T. microellipsoides* Histone H3 reverse	CACGCTTAGCATGAATAGCAG
CB299	*T. microellipsoides* EFG1 knockout construct forward	TACAGGGATCCGCTGTGTAGGGTGCTTCAT
CB300	*T. microellipsoides* EFG1 knockout construct reverse	GTAGCAGGTACCCAACTTCCCTCGTAATGA TCC
CB301	*T. microellipsoides* EFG1 knockout backbone primer forward	GGAGGGTATTCTGGGCCTCCATGTCCGCCA TAACTCCATCATAAGATGGC
CB302	*T. microellipsoides* EFG1 knockout backbone primer reverse	TATGTGAATGCTGGTCGCTATACTGAAACCT TTGACAATATTTTGAAGAC
CB303	*T. microellipsoides* EFG1 KanMX middle forward	GCCATCTTATGATGGAGTTATGGCGGACAT GGAGGCCCAGAATACCCTCC
CB304	*T. microellipsoides* EFG1 KanMX middle reverse	GTCTTCAAAATATTGTCAAAGGTTTCAGTAT AGCGACCAGCATTCACATA
CB307	*T. microellipsoides* EFG1 KanMX integration check forward	CTGACATTGACCCTCCAAAG
CB025	KanMX integration check forward	CCCATATAAATCAGCATCCATG
CB026	KanMX integration check reverse	TATGGTATTGATAATCCTGATATG
CB280	*T. microellipsoides* EFG1 qPCR forward	**GTAAACTGAGCAGGAATCGG**
CB281	*T. microellipsoides* EFG1 qPCR reverse	GTAACATGCCCAATAGACAAAC
CB493	*T. microellipsoides* CTT1 qPCR	GGTCCAAGGGGTACTCCG

	forward	
CB494	*T. microellipsoides* CTT1 qPCR reverse	CCATTCTCAGGACGGACATG
CB495	*T. microellipsoides* GAP1 qPCR forward	GGTGGTGCCATTGGTACTG
CB496	*T. microellipsoides* GAP1 qPCR reverse	CACCAACAGACATCACCATACAG
CB497	*T. microellipsoides* GLK1 qPCR forward	GGAGCTGTTGAGAAATATGCTTG
CB498	*T. microellipsoides* GLK1 qPCR reverse	TGTGCGATAAAACCTCACTCG
CB499	*T. microellipsoides* MEP2 qPCR forward	CCACATTCCGTTACCTCAGTT
CB500	*T. microellipsoides* MEP2 qPCR reverse	GTGTTCATAGCAGAATACCAAGC
CB501	*T. microellipsoides* SSA3 qPCR forward	GAGAGCATTGTCCTCGTCTTC
CB502	*T. microellipsoides* SSA3 qPCR reverse	GTCGATCTGAACATGTCAGCAC
CB503	*T. microellipsoides* RPL30 qPCR forward	GTTGGCTTTAGTTGTCAAGTCTG
CB504	*T. microellipsoides* RPL30 qPCR reverse	CGGACTTTCTCAACACTGGG
CB507	*T. microellipsoides* SOD2 qPCR forward	CCTATGTCAATGGTTTCAACACTG
CB508	*T. microellipsoides* SOD2 qPCR reverse	GTAAAACCACCACCATGAAACTTC
CB510	*T. microellipsoides* XRN1 qPCR forward	GAGGTTCTGTTGGTGCCGAC
CB511	*T. microellipsoides* XRN1 qPCR reverse	TCAACATCTGGCATTTGGTACTC

**Strains and Plasmids**		
**Strain name**	**Use**	**antibiotic marker**
Torulaspora microellipsoides WT (NCYC 2568) white cells	WT white cells	none
Torulaspora microellipsoides WT (NCYC 2568) opaque cells	WT opaque cells	none
Torulaspora microellipsoides WT (NCYC 2568) Δefg1	WT white cells with *efg1* deletion	KanMX (G418)
**Plasmid name**	**Use**	**antibiotic marker**
pUC19_Tmicro_efg1	cloning of EFG1 ORF +/- 1kb	Ampicillin
pUC19_tmicro_efg1KanMX	deletion of EFG1 with ORF replaced with KanMX	Ampicillin, KanMX (G418)

76
