# Supplementary material for: A white-to-opaque-like phenotypic switch in the yeast Torulaspora microellipsoides
Source: Commun Biol. 2020 Feb 28;3:86. doi: 10.1038/s42003-020-0815-6 (PMC7048803; doi:10.1038/s42003-020-0815-6)
Supplement: Supplementary file 2 — Description of additional supplementary items [file 42003_2020_815_MOESM2_ESM.pdf]

Description of additional supplementary items

The Supplementary Data 1 excel file contains the results of all RNA-seq analyses, as well as all raw data values used to generate figures in which data values are shown. The first two sheets show genes differentially regulated between white and opaque cells with relevant expression values and statistics, as well as analyses used to generate the pie charts shown in Figure 3, while the third tab specifically shows transcriptional factors differentially expressed between the two cell types. Sheet 4 shows the mapping efficiencies and average sequencing depth for the RNA seq libraries. Sheets 5-12 contain all raw data for Figure 2, Figure 4, Figure 5, and all supplemental figures in which numerical data points are plotted. All sheets are labelled clearly.
