# Supplementary material for: A white-to-opaque-like phenotypic switch in the yeast Torulaspora microellipsoides
Source: Commun Biol. 2020 Feb 28;3:86. doi: 10.1038/s42003-020-0815-6 (PMC7048803; doi:10.1038/s42003-020-0815-6)
Supplement: Supplementary file 5 — Reporting Summary [file 42003_2020_815_MOESM5_ESM.pdf]

nature research

Corresponding author(s): Matthew Dahabieh

Last updated by author(s): Jan 10, 2020

# Reporting Summary

Nature Research wishes to improve the reproducibility of the work that we publish. This form provides structure for consistency and transparency in reporting. For further information on Nature Research policies, see Authors & Referees and the Editorial Policy Checklist.

## Statistics

For all statistical analyses, confirm that the following items are present in the figure legend, table legend, main text, or Methods section.

| n/a | Confirmed | |
|---|---|---|
| ☐ | ☒ | The exact sample size ($n$) for each experimental group/condition, given as a discrete number and unit of measurement |
| ☐ | ☒ | A statement on whether measurements were taken from distinct samples or whether the same sample was measured repeatedly |
| ☐ | ☒ | The statistical test(s) used AND whether they are one- or two-sided<br>*Only common tests should be described solely by name; describe more complex techniques in the Methods section.* |
| ☐ | ☒ | A description of all covariates tested |
| ☐ | ☒ | A description of any assumptions or corrections, such as tests of normality and adjustment for multiple comparisons |
| ☐ | ☒ | A full description of the statistical parameters including central tendency (e.g. means) or other basic estimates (e.g. regression coefficient) AND variation (e.g. standard deviation) or associated estimates of uncertainty (e.g. confidence intervals) |
| ☐ | ☒ | For null hypothesis testing, the test statistic (e.g. $F$, $t$, $r$) with confidence intervals, effect sizes, degrees of freedom and $P$ value noted<br>*Give P values as exact values whenever suitable.* |
| ☒ | ☐ | For Bayesian analysis, information on the choice of priors and Markov chain Monte Carlo settings |
| ☒ | ☐ | For hierarchical and complex designs, identification of the appropriate level for tests and full reporting of outcomes |
| ☒ | ☐ | Estimates of effect sizes (e.g. Cohen's $d$, Pearson's $r$), indicating how they were calculated |

*Our web collection on statistics for biologists contains articles on many of the points above.*

## Software and code

Policy information about availability of computer code

| | |
|---|---|
| Data collection | DNA seq, RNA seq, transcripts annotation and expression levels were deposited on Gene Expression Omnibus (accession: GSE136991) and are freely available. |
| Data analysis | All software used for data processing and analysis was referenced in the manuscript. |

For manuscripts utilizing custom algorithms or software that are central to the research but not yet described in published literature, software must be made available to editors/reviewers. We strongly encourage code deposition in a community repository (e.g. GitHub). See the Nature Research guidelines for submitting code & software for further information.

## Data

Policy information about availability of data

All manuscripts must include a data availability statement. This statement should provide the following information, where applicable:

- Accession codes, unique identifiers, or web links for publicly available datasets
- A list of figures that have associated raw data
- A description of any restrictions on data availability

All raw values for Figures 1, 4, 5, Supplementary 3, Supplementary 7, Supplementary 8, Supplementary 10, and Supplementary 12 are given in Supplementary Data 1. Raw sequence data has been deposited on GEO (GSE136991).

# Field-specific reporting

Please select the one below that is the best fit for your research. If you are not sure, read the appropriate sections before making your selection.

☒ Life sciences ☐ Behavioural & social sciences ☐ Ecological, evolutionary & environmental sciences

For a reference copy of the document with all sections, see nature.com/documents/nr-reporting-summary-flat.pdf

# Life sciences study design

All studies must disclose on these points even when the disclosure is negative.

| | |
|---|---|
| Sample size | In all experiments, measurements were taken from at least three independent cultures of yeast, or in the case of dilution plating assays, experiments were performed at least three independent times. |
| Data exclusions | No data was excluded. |
| Replication | Multiple independent experiments were performed with identical or highly similar results every time, thus ensuring reproducibility. With regard to RNA-seq data, experiments thereafter in the paper such as RT-qPCR of selected genes or phenotypic outputs strongly support the reproducibility of this data set. |
| Randomization | Not relevant to study -- model organism, yeast. |
| Blinding | This study includes only analytical measurements of specific samples or phenotypes, thus blinding is not required. |

# Reporting for specific materials, systems and methods

We require information from authors about some types of materials, experimental systems and methods used in many studies. Here, indicate whether each material, system or method listed is relevant to your study. If you are not sure if a list item applies to your research, read the appropriate section before selecting a response.

## Materials & experimental systems

| n/a | Involved in the study | |
|---|---|---|
| ☒ | ☐ | Antibodies |
| ☒ | ☐ | Eukaryotic cell lines |
| ☒ | ☐ | Palaeontology |
| ☒ | ☐ | Animals and other organisms |
| ☒ | ☐ | Human research participants |
| ☒ | ☐ | Clinical data |

## Methods

| n/a | Involved in the study | |
|---|---|---|
| ☒ | ☐ | ChIP-seq |
| ☐ | ☒ | Flow cytometry |
| ☒ | ☐ | MRI-based neuroimaging |

## Flow Cytometry

### Plots

Confirm that:

☒ The axis labels state the marker and fluorochrome used (e.g. CD4-FITC).

☒ The axis scales are clearly visible. Include numbers along axes only for bottom left plot of group (a 'group' is an analysis of identical markers).

☒ All plots are contour plots with outliers or pseudocolor plots.

☒ A numerical value for number of cells or percentage (with statistics) is provided.

### Methodology

| | |
|---|---|
| Sample preparation | Standard preparation for DNA content analysis in yeast. Fix cells in ethanol, treat with RNaseA, add DNA stain (sytox green), run on flow cytometer. |
| Instrument | Attune NxT Flow Cytometer |
| Software | FloJo |
| Cell population abundance | Singlets were separated from doublets. |

Gating strategy

Doublet discrimination SSC-H vs SSC-W, then SSC-A vs SSC-H, then cell gate SSC-A vs FSC-A. Thereafter, a histogram of cell number vs Sytox Green (FITC channel) is displayed.

☒ Tick this box to confirm that a figure exemplifying the gating strategy is provided in the Supplementary Information.
